# Efficacy of Residential, Group-Based, Intensive Holistic Lifestyle Intervention Among Type-2 Diabetes Patients - A Single Group Pre- And Post-intervention Study

**DOI:** 10.7759/cureus.22253

**Published:** 2022-02-15

**Authors:** Sahu Shrimant Kumar, M Santhi Sree, Mohandas Manjusha, Murali Mohan Reddy, Ramya B, Bala Kishore, Rohini sharma G, Jagat Jit Mohapatra

**Affiliations:** 1 Diabetology and Holistic Care, J. Watumull Global Hospital and Research Centre, Mount Abu, IND; 2 Community Medicine, Ananta Institute of Medical Sciences and Research Centre, Rajsamand, IND; 3 Clinical Psychology, National Institute of Mental Health and Neurosciences, Bangalore, IND; 4 Community Medicine, Evidencian Research Associates, Bangalore, IND; 5 Diabetology, Dr. Mohan's Diabetes Specialities Centre, Chennai, IND; 6 Vedic Sciences, MIT Art Design and Technology University, Pune, IND; 7 Evidencian Science, Evidencian Research Associates, Bangalore, IND; 8 Diabetology, J. Watumull Global Hospital and Research Centre, Mount Abu, IND

**Keywords:** body mass index, non-communicable diseases, holistic exercise, therapeutic lifestyle modifications, glycemic control, type 2 diabetes

## Abstract

Background

The present study assessed the efficacy of the residential, group-based, intensive holistic lifestyle intervention on glycaemic control.

Materials and methods

A one-group pre and post-intervention study was conducted among 145 people with diabetes for a period of one year from February 2019 to January 2020. The study population underwent "Brahma Kumaris Raja Yoga lifestyle" intervention. Outcome variables were changes in HbA1c levels and anthropometric parameters (like weight, BMI, etc.). Paired t-test was used to compare normally distributed numeric variables.

Results

The mean age was 52.39±5.79 years, with a male-female ratio close to 1:1. Mean HbA1c at baseline was 9.06±2.1%. The mean weight and BMI were 71.03±12.84 kg and 28.28±4.83kg/m^2^, respectively. Mean HbA1c value had shown a reduction of 1.60% (95%CI 1.17 to 1.90, p <0.001) at three months and 1.58% (95% CI 1.13-1.87, p<0.001) reduction at a six-month follow-up. Between the third and sixth months, there was no significant change in the HbA1c value. Mean weight reduced by 0.79 kg (95% CI 0.08-1.08, p=0.023) at six-month follow-up and mean BMI decreased by 0.31 units from baseline to three months (95% CI 0.05-0.56, p=0.017). A statistically significant reduction was observed in waist circumference at the third month (MD=1.61 95% CI =0.95 to 2.28, P<0.001) and sixth month (MD=1.53, 95% CI 0.82-2.25, p<0.001) follow-up.

Conclusion

This residential, group-based, intensive holistic lifestyle intervention showed a significant decrease in HbA1c levels and anthropometric parameters at three- and six-months follow-up, thereby improving the overall health and wellbeing of people with diabetes.

## Introduction

Diabetes has become a global pandemic leading to 1.5 million deaths each year, accounting for 10% of the global all-cause mortality [1]. Currently, 500 million people worldwide are diabetics, and by 2045, a 30% increase in the present number can be expected, as estimated by the International Diabetes Federation (IDF). Surprisingly, an almost equal number of people suffer from impaired glucose tolerance (IGT), which is considered as a predecessor to an even worse diabetes pandemic in the future [2]. India has become the country with the second-largest diabetes population, with one in six adults with diabetes in the world [3,4]. According to IDF, An estimated 77 million Indians live with diabetes, with a prevalence of 8.9%, leaving a wider impact on global health, economy, and society [5].

To prevent the complications and progression of diabetes, American Diabetes Association (ADA) guidelines have recommended a variety of anti-diabetic agents along with an intensive behavioral lifestyle intervention program [6,7]. To predict better outcomes among type 2 diabetes mellitus (T2D) patients, seven essential self-care behaviors are formulated based on healthy eating, being physically active, monitoring of blood sugar, compliance with medications, good problem-solving skills, healthy coping skills, and risk-reduction behaviors [8]. There is unequivocal evidence documenting favorable therapeutic outcomes with interventions providing enough emphasis on these self-care practices [9]. 

Emphasis on diabetes self-management through trained primary practitioner or educators have been proven to overcome negative habits and promote diabetes self-management behaviors [10]. But emphasis provided on the non-pharmacological lifestyle components is quite variable. There is a need to develop approaches in increasing self-care practice, as a systematic review and meta-analysis reported poor self-care practice among type 2 diabetes mellitus (T2DM) patients [11]. Previous reviews and meta-analyses have reported that both lifestyle modification (LSM) and medications are beneficial in preventing progression to diabetes. Data on modalities offering long-term efficacy in glycaemic control remain discordant, as there are inconsistent results regarding the type, frequency, and intensity of LSM or medications [12]. Even when the self-care components are incorporated in treatment protocols, the time and emphasis provided on them are often inadequate, especially in outpatient settings.

Another key concern is not providing enough emphasis on the involvement of family members in the care process. Compared with the individual format, the group format involving family, friends have been demonstrated to be more empowering to diabetic patients [13].

Families can be an instrument for emotional support, as people with T2DM are snuggled around their families and large social environment. Involving the entire family is reported to improve the T2DM individual's care and helps prevent the risk of developing T2DM in the family members [14]. Family support can improve self-management in controlling glycaemic levels among T2D patients and can be a very good direction to improve diabetes care [15]. But a majority of the interventions do not provide due emphasis on the involvement of family members to build an enabling environment to effectively practice self-care behaviors.

It has been reported that worldwide 30-57% of the population is dissatisfied with conventional medical management of diabetes and often turn to alternative other alternative modalities of therapy [16]. To enhance physical health, a traditional system named yoga originated in India over 4000 years ago. Two systemic reviews and meta-analyses have shown that an integrated yoga lifestyle is a safe and effective intervention in adults with type 2 diabetes [17,18]. India is a country that is steeped in tradition and boasts a rich history of healing practices, and 67% diabetic population follow complementary and alternative medicines (CAMs) [19].

Of the several significant branches of yoga in India, the most widely practiced forms include Raja Yoga. Brahma Kumaris (BK) teach Raja Yoga meditation along with associated lifestyle changes. A pilot study has already tried this BK meditation technique on many patients attending their meditation centers, and remarkable changes in emotions, behavior, and glycaemic control were observed. Considering the strong emphasis on holistic behavioral change, traditional outpatient clinic-based approach, just focusing on the patient alone may not be effective. However, the feasibility and impact of delivering a composite intervention through a residential program; closely involving the family members in promoting self-care practices among the diabetic population is not studied. The present study aimed to assess the effectiveness of the residential, group-based, intensive holistic Brahma Kumari's Raja Yogi lifestyle intervention on glycaemic control among type 2 diabetes patients.

Aims and objectives

The present study assessed the efficacy of the residential, group-based, intensive holistic lifestyle intervention on glycaemic control. It also aimed to assess the impact of the intervention on anthropometric parameters, including weight, BMI, and waist circumference at three months and six months follow-up.

## Materials and methods

The present study was a one-group pre-test and post-test study conducted among type 2 diabetics attending the community center for lifestyle disease management interventions between February 2019 to January 2020. The study was registered as per The Clinical Trials Registry- India (CTRI) guidelines. The study was approved by the community center's research and ethical committee membersF. Written informed consent was obtained from participants, and confidentiality of participants was maintained throughout the study.

**Figure 1:**
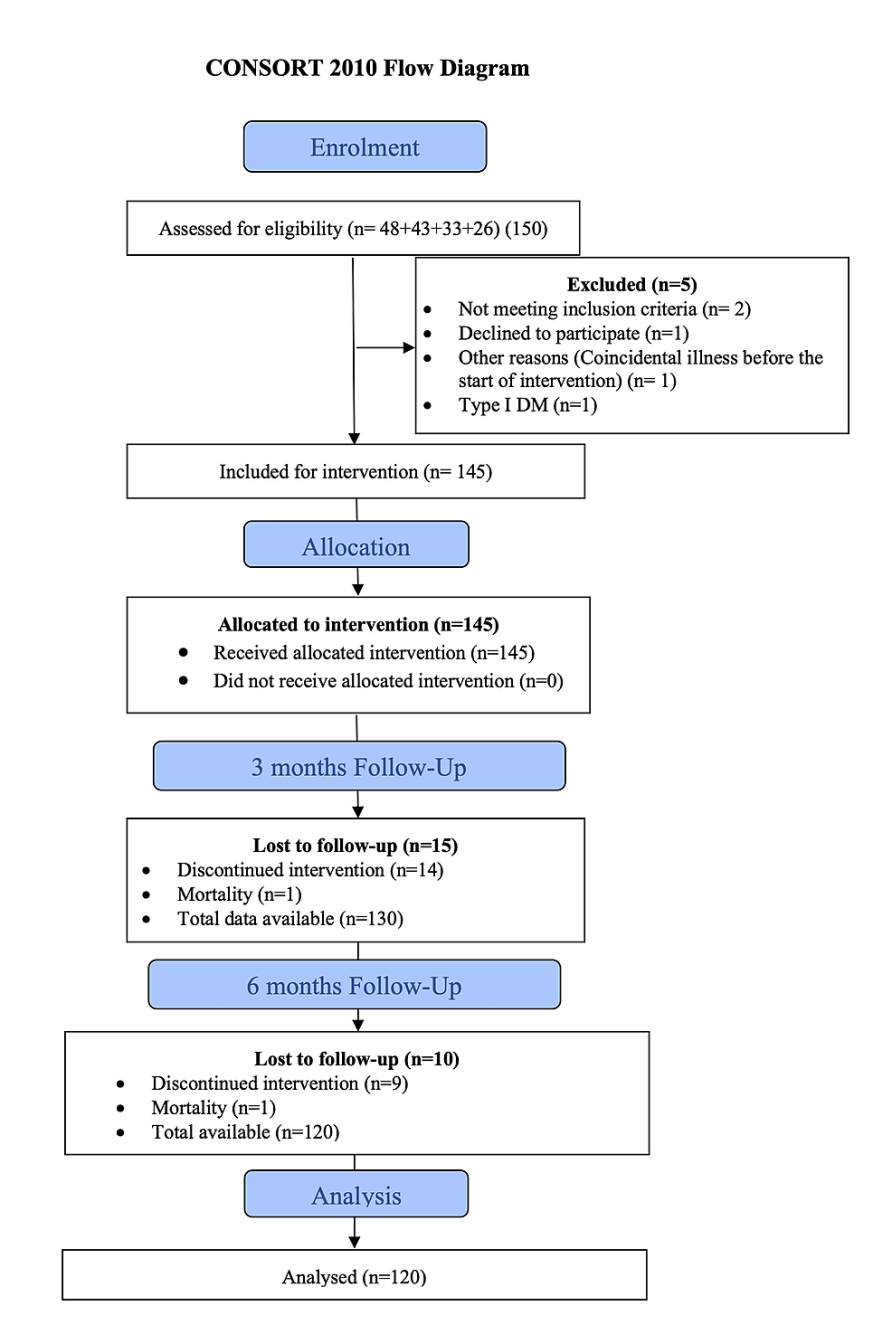
Flow diagram showing the flow of participants through the intervention according to criteria recommended by CONSORT guidelines CONSORT - Consolidated Standards of Reporting Trials

Figure 1 depicts a flow diagram showing the flow of participants through the intervention according to criteria recommended by Consolidated Standards of Reporting Trials (CONSORT) guidelines.

Sample size and sampling technique

The required number of participants was selected by simple random sampling from the master list. The sample size was calculated assuming the expected mean HbA1c value in the study population as per Solanki JD et al. [20] as 7.95±1.89, which was conducted on people from the same geographical locality current study population. To document the 0.95-unit reduction in HbA1c level to 7% (American Diabetes Association cut-off level for reasonable glycaemic control), the required number of subjects would be 122. To account for a loss to follow-up of about 10%, 145 participants were included. Table 1 shows the inclusion and exclusion criteria of the study population.

**Table 1:** Inclusion and exclusion criteria of the study population

Inclusion criteria:	Exclusion criteria:
Both male and female participants with type 2 diabetes mellitus	Major Psychiatric disorders, including schizophrenic patients
Age between 30 to 60 years.	Pregnant and lactating woman.
Body mass index of > 27.0kg/m^2^	People with an established microvascular complication like proliferative diabetic retinopathy, renal failure (acute or chronic), type 1 diabetes, severe osteoarthritis (OA)
	Established cases of coronary artery disease (CAD) and cardiovascular diseases

Data collection

All the participants were interviewed by qualified clinical psychologists to assess their psychological wellbeing. The participants were trained, examined, and monitored by a team of qualified medical practitioners and clinical psychologists led by the principal investigator. After thoroughly explaining the study procedures, nature of the interventions, possible risks, and benefits, all the study participants were recruited.

Investigations

All participants underwent a thorough clinical and physical examination. About 5 ml of venous blood was collected at the beginning of the study and was sent to the laboratory under proper transport conditions for the assessment of plasma blood glucose, HbA1c, at the baseline. Periodical blood sugar monitoring was done six times a day with a glucometer (i-SENS NoCoding 1 Plus Glucometer, i-SENS Inc., Seoul, South Korea) during the days of the intensive residential program.

Intervention

It was a residential-based intervention. Participants with one family member were supposed to stay in the community center throughout the intervention period. The study group was given a specific "Brahma Kumari's Raja Yogi lifestyle" intervention in their daily life for six months. The intervention has four steps.

Balanced, Vegetarian, and Pure Diet

Depending upon the height, weight, age, and gender, a balanced diet (around 1500-2000 calories per day, diet plan varied from person to person depending on height and weight) contains amino acids, vitamins, minerals, fats, carbohydrates, and other nutrients to maintain health, vitality, and general wellbeing. This diet also provides extra nutrients to withstand the short duration of illnesses.

Walking Yoga and Holistic Exercise

A simple walking exercise practiced indoor/outdoor involves the whole body (morning 30 minutes, evening 30 minutes, burning around 400-500 calories). The holistic exercise combines aerobic, anaerobic flexibility, breathing and relaxation techniques, addressing complete physical body, emotional health, and spiritual states.

The above two interventions are for the physical dimension of health.

Raja Yoga Meditation

Purely a mental procedure and not a traditional yoga (postural exercises and asanas). The practitioner is encouraged to wake up early, and a Guided Commentary of powerful positive thoughts is given, which is to be experienced through visualization. There is no mantra to chant or words to cram and remember. Practitioners are explained about the Supreme Being the source of tremendous positive powers and asked to visualize and experience the powerful rays coming down from the Supreme and charging the entire physical body that helps in hyperglycemia and other physiological derangements getting corrected.

Positive Thinking

Special training is given to create positive thoughts which are creative, powerful, purposeful, pure, and divine for self-empowerment.

The last two interventions are mainly for mental and spiritual dimensions of health.

Post-intervention

The participants were followed up after three months and finally evaluated after completing six months of intervention.

Study variables

Changes in HbA1c levels were considered as the primary outcome variable. Changes in anthropometric parameters (like weight, BMI, etc.) at three and six-month follow-up were considered as secondary outcome variables.

Statistical Methods

Quantitative variables like age, height, weight, BMI, hip and waist circumference, duration of diabetes were presented as mean and standard deviation. Categorical variables like marital status, education other life-related characteristics were presented as frequency and proportion. The change in the quantitative parameters before and after the intervention was assessed by paired T-test (in case of two time periods) or one-way repeated measures ANOVA (in case of comparison across more than two time periods). P-value ≤0.05 was considered statistically significant. R studio (RStudio, Boston, Massachusetts) and coGuide version V.1.0 was used for statistical analysis [21].

## Results

A total of 145 subjects were included in the final analysis.

Baseline data

The baseline characteristics of the study population are described in Table 2. The mean age of the study participants was 52.39± 5.79 years, with a male-female ratio close to 1:1. The mean duration of diabetes was 7.41± 6.39 years. Majority of 84 (57.9%) participants were diagnosed with diabetes during their routine check-up for other illnesses. Among the participants, 114 (78.6%) had obesity, 53 (36.6%) had hypertension, and 50 (34.5%) had dyslipidemia.

**Table 2:** Demographic and health information of the study population (N=145)

Parameter	Frequency
Age in years (mean±SD)	52.39± 5.79 (range 35 to 60)
Gender (Male: female ratio)	73:72
Marital status	
Single	4 (2.8%)
Married	135 (93.1%)
Widower/ Widow/ Divorced	6 (4.1%)
Education	
Illiterate	4 (2.76%)
Middle school	13 (8.97%)
High school	37 (25.52%
Higher secondary	20 (13.79%)
Graduate	51 (35.17%)
Literate	3 (2.07%)
Postgraduate and professional	17. (11.72%)
Religion	
Hindu	144 (99.31%)
Muslim	1 (0.69%)
Locality	
Urban	139 (95.86%)
Rural	6 (4.14%)
Type of family	
Nuclear	106 (73.10%)
Joint	37 (25.52%)
Extended	2 (4.14%)
Duration of diabetes in years (mean±SD)	7.41± 6.39 (range 0.08 to 28)
Mode of onset	
Acute	14 (9.7%)
Sub-acute	23 (15.9%)
Insidious	108 (74.5%)
How was diabetes diagnosed	
Accidentally	18 (12.4%)
While health screening	40 (27.6%)
With complications of diabetes mellitus	3 (2.1%)
Routine check-ups for other illnesses	84 (57.9%)
Co-morbid conditions	
Obesity	114 (78.6%)
Hypertension	53 (36.6%)
Dyslipidaemia	50 (34.5%)
Hypothyroidism	22 (15.2%)
Cataract	11 (7.6%)

Three (2.1%) of the participants were smokers, and seven (4.8%) participants consumed alcohol. A sedentary lifestyle was reported among 120 (82.8%) participants. The majority of the current study population were vegetarians and reported having more than three meals per day (Table 3).

**Table 3:** Lifestyle-related variables of the study population (N=145)

Parameter	Frequency
Smoker	
Current	3(2.1%)
Past	8(5.5%)
Never	134(92.4%)
Consumes alcohol	
Current	7(4.8%)
Past	4(2.8%)
Never	134(92.4%)
Type of work/occupation	
Sedentary	120(82.8%)
Light outdoor	21(14.5%)
Heavy outdoor	4(2.8%)
Dietary preference	
Mixed vegetarian and nonvegetarian	8(5.6%)
Vegetarian	137(94.5%)
Number of main meals per day	
Two times	41(28.3%)
Three times or more	104(71.8%)

Clinical changes at three and six months follow-up

The primary and secondary outcome parameters before and after intervention are summarized in Table 4. Fifteen subjects lost follow-up (discontinued intervention [n=15]; mortality [n=1]) at three months reducing the sample size to 130 and another 10 subjects lost follow-up (discontinued intervention [n=9]; mortality [n=1]) at six months further reducing the sample size to 120. Hence, 130 subjects were analyzed at three months, and 120 subjects were analyzed at a six-month follow-up.

**Table 4:** Changes in HbA1c levels and anthropometric parameters at baseline and after the third and sixth month

Parameter	Baseline (N=145) mean ± SD	Third month (N=130) mean ± SD	Sixth month (N=120) mean ± SD
Primary outcome parameters			
HbA1c (%)	9.06±2.1	7.46±1.43	7.48±1.35
Secondary outcome parameters			
Weight (in Kg)	71.03 ± 12.84	71.08 ± 14.82	70.35 ± 12.73
Body mass index	28.28 ± 4.83	28.02 ± 4.78	28.06 ± 4.78
Waist circumference (in cm)	95.31 ± 9.82	93.65 ± 10.07	93.45 ± 10.24
Hip circumference (in cm)	102.53 ± 10.72	99.92 ± 10.65	99.71 ± 10.85
Waist to hip ratio	0.92 ± 0.11	0.93 ± 0.07	0.93 ± 0.07

Mean HbA1c at baseline was 9.06 ±2.1 % at baseline. At the three-month follow-up period, there was a 1.60% reduction (95% CI 1.17 to 1.90, p<0.001) in the HbA1c value from the baseline. At the six-month follow-up period, there was a 1.58% (95% CI 1.13 to 1.87, p<0.001) reduction in HbA1c value from the baseline. No statistically significant change in the HbA1c value was observed between the third and sixth months. At the baseline, the mean weight and body mass index were 71.03 ± 12.84 kg and 28.28 ± 4.83, respectively. The mean waist circumference was 95.31 ± 9.82 cm. There was a 0.79 kg reduction in the mean weight at six months (95% CI 0.08- 1.08, p=0.023). A 0.31 unit decline was noted in the body mass index from baseline to three months (95% CI 0.05 to 0.56, p=0.017). But by the end of six months, the observed difference in body mass Index from baseline was only 0.28 units (95% CI -0.56 to 0.53, p=0.11). Also, a statistically significant reduction was observed in waist circumference at the third month (MD=1.61 95% CI =0.95 to 2.28, p<0.001) and sixth month (MD=1.53, 95% CI 0.82 to 2.25, p<0.001) follow up periods (Table 5).

**Table 5:** Comparison of the primary and secondary outcome parameters at baseline, third month, and sixth month following the intervention

	Baseline vs. third month (n=130)		Baseline vs. sixth month (n=120)		Third month vs. sixth month (n=120)	
	Mean difference (95 % CI)	p-value	Mean difference (95 % CI)	p-value	Mean difference (95 % CI)	p-value
Primary outcome parameters						
HbA1c (%)	1.60 (1.17- 1.90)	<0.001	1.58 (1.13-1.87)	<0.001	0.02 (0.09-0.30)	0.294
Secondary outcome parameters						
Weight (in kg)	0.06 (-1.51- 1.61)	0.946	0.79 (0.08- 1.08)	0.023	0.73 (-1.09-2.22)	0.503
Body mass index	0.31 (0.05- 0.56)	0.017	0.28 (-0.56 -0.53)	0.112	0.04 (-0.06- 0.22)	0.148
Waist circumference (in cm)	1.61 (0.95 – 2.28)	<0.001	1.53 (0.82 – 2.25)	0.001	0.20 (-0.22- 0.58)	0.572
Hip circumference (in cm)	2.82 (2.13-3.51)	<0.001	3.02 (0.47- 2.08)	<0.001	0.21 (-0.55-0.82)	0.696
Waist to hip ratio	0.01 (0.003-0.02)	0.135	0.01 (0.003-0.03)	0.111	0 (0.005-0.001)	0.332

## Discussion

This study from India, which focused on the residential-based involvement of family members in decreasing glycemic and anthropometric parameters among diabetes by providing a specific "Brahma Kumari's Raja Yogi lifestyle" intervention. A statistically significant reduction was observed in mean HbA1c value, weight, mean BMI, and waist circumference at the third month (p<0.001) and sixth month (p<0.001) follow-up. 

Various types and forms of yoga-based interventions are gaining momentum in recent years. A methodological study by Nagarathna et al. [22] had reported the implementation of a nationwide multicentric study consisting of a validated culturally acceptable yoga-based lifestyle intervention called "*niyantrita madhumeha bharata abhiyaan*". A large number of diabetic and nondiabetic populations were enrolled for this cluster randomized controlled trial. Among the diabetic population enrolled for the study, the HbA1c levels were 7.63±2.17 and 7.86±2.13 in the intervention and control groups, indicating poor glycaemic control. This indicates the strong willingness of the diabetic population with sub-optimal glycaemic control to adapt alternative interventions. The mean age in the present study was 52.39 ±5.79 years which is in contrast to a systematic review on Feel4Diabetes school and community-based intervention by Kivela et al. [23], where participants' mean age was <45 years. 

In the present study, the mean HbA1c at baseline was 9.06±2.1% that showed a reduction of 1.60% (95%CI 1.17 to 1.90, p<0.001) at three months and 1.58 % (95% CI 1.13-1.87, p<0.001) reduction at the six-month follow-up. The mean BMI decreased, 0.31 units from baseline to three months (95% CI 0.05-0.56, p=0.017). The findings were similar to the family functional-based coaching program by Pamungkas et al. [24], where they showed a positive decline in glycated hemoglobin (pre-test- 8.0 ±1.9: post-test 6.4±1.1%) and total cholesterol levels after receiving the 12-week program, and there was no significant difference found in body mass index.

Developing diabetes interventions with family support should be an integral part of sustaining self-management behaviors and improving the health outcomes of T2D patients. The results confirmed the impact of family integration on several health outcomes of T2D. To effectively engage family members in the intervention, a clear understanding of the theoretical basis of involving family members is needed to serve the T2D patients in changing behaviors. A multinational survey shows that only 25% of family members attended a diabetes program. This low participation of family members becomes a hindrance in developing a chronic illness support model in controlling diabetes [25]. A study by Mayberry et al. [26] found that using Family-Focused Add-On for Motivating Self-Care (FAMS) for two weeks increased self-care and improved support for and communication about diabetes. In contrast, Mayberry et al. [27] found involvement of untaught family members compromised patients' self-care and glycaemic control.

Although physical activity and nutrition represent a cornerstone for managing T2D, it is often difficult to incorporate regular physical activity into daily lives in combination with healthy nutritional intake. This can be made possible with the help of community /group-based residential interventional programs. Community-based residential interventions can deliver culturally appropriate health education which can improve self-care compliance and adherence to self-management practices. These interventions are cost-effective and practical and provide long-term benefits to a larger section of people in need of such interventions.

A systematic review with the meta-analysis by Plotnikoff et al. [28] demonstrated community-based physical activity interventions in significantly lowering of HbA1c levels by −0.32% (95% CI −0.65, 0.01, p<0.06). A qualitative study by Morrison et al. [29] found that community-based participatory learning and action (PLA) interventions are an effective and cost-effective approach to addressing diabetes in rural Bangladesh. Lancers et al. [30], in their three-week lifestyle intervention residential program for T2D individuals, found a reduction in medication costs. The medication cost was reduced as there was better glycaemic control due to high exercise volume, diet restriction, and health education.

The findings of the study showed that residential, group-based, intensive, holistic lifestyle intervention significantly decreased HbA1c levels and anthropometric parameters at three- and six-months follow-up. The provision of this lifestyle intervention could allow a large proportion of individuals with diabetes to achieve improvements in critical cardio-metabolic outcomes, with potential long-term benefits for health and wellbeing. Brahma Kumaris Raja Yoga lifestyle incorporated a vegetarian diet, targeting the elevated glycaemic and lipid levels, ultimately reducing the micro and macrovascular complications of diabetes. These low-cost and safe methods of lifestyle interventions can contribute to reducing the severity of diabetic comorbidities.

Considering the composite nature of the intervention, the level of adherence to various components of the intervention is an important determinant of the outcome. Documenting and quantifying the level of adherence was particularly challenging. Many participants failed to fill the daily activity diaries on regular basis with complete details. Hence attributing the changes in the outcome parameters to different components of intervention was not possible. Difficulties in documenting the impact of the intervention on key lifestyle parameters like diet and physical activity etc. was another major challenge encountered. Another key limitation of the study is the absence of a control group, precluding us from making any conclusions about the relative superiority of the current intervention as compared to the existing standard of care. The majority of the current study population was vegetarians, and the prevalence of smoking and alcoholism reported is considerably low in the study population. Hence generalizability of the current study findings is limited to population groups with similar lifestyle patterns. Another limitation is that we only reported outcomes at three and six months. Even if diabetes gets reversed in later stages, the clinically significant glycaemic control achieved will contribute to reducing microvascular complications with the help of metabolic memory. Difficulty in maintaining the long-term high volume of intervention data could be one of the pitfalls of such interventions and may require a revised prescription of instructions at regular intervals. Similar studies in the future are needed to investigate the long-term changes in HbA1c levels and how the change can be maintained throughout with the help of some booster sessions. The effectiveness of intervention in modulating behavior change needs to be studied further.

## Conclusions

Despite the limitations specified above, the current study has categorically demonstrated that a group-level intervention, with the involvement of the family members delivered in short term residential setting, is feasible. Also, minimal dropout rates from the study during the two-week residential phase indicate high acceptance levels for the intervention. 

Composite interventions, with shifting the balance of emphasis to non-pharmacological components, involvement of family members is gaining momentum in diabetes care. Time-tested practices like "Brahma Kumari's Raja Yogi lifestyle" perfectly complement pharmacological therapy and have the potential to improve physical, psychological, and spiritual wellbeing. Hence there is a strong need to conduct further studies to document the feasibility and impact of this intervention in different settings. Scaling up such complementary interventions can result in a huge positive impact on the diabetic population, their families, and society. Also, there is a strong need to identify suitable procedures to document the entire spectrum of challenges and benefits of such interventions is also essential. Appropriate health economic assessment to document cost-effectiveness and cost-benefit of such interventions is also needed.
